# Novel cytoplasmic lncRNA IKBKBAS promotes lung adenocarcinoma metastasis by upregulating IKKβ and consequential activation of NF-κB signaling pathway

**DOI:** 10.1038/s41419-021-04304-4

**Published:** 2021-10-26

**Authors:** Yuanxin Xing, Yani Lin, Ying Zhang, Jing Hu, Junmei Liu, Yuanyuan Tian, Jian Zhao, Weiwen Chen, Bo Han

**Affiliations:** 1 Department of Biochemistry and Molecular Biology, School of Basic Medical Sciences, Cheeloo College of Medicine, Shandong University, Jinan, 250012 Shandong China; 2 Department of Pathology, Qilu Hospital of Shandong University, Jinan, 250012 Shandong China; 3 Department of Thoracic Surgery, Qilu Hospital of Shandong University, Jinan, 250012 Shandong China; 4 The Key Laboratory of Experimental Teratology, Ministry of Education and Department of Pathology, School of Basic Medical Sciences, Cheeloo College of Medicine, Shandong University, Jinan, 250012 Shandong China

**Keywords:** Cancer epidemiology, Lung cancer

## Abstract

NF-κB signaling pathway is a critical link between inflammation and cancer. Emerging evidence suggested that long non-coding RNAs (lncRNAs) were involved in dysregulation of NF-κB. Herein, we reported a novel lncRNA IKBKBAS that activated NF-κB in lung adenocarcinoma (LUAD) by upregulating IKKβ, a key member of NF-κB signaling pathway, thereby promoting the metastasis of LUAD both in vitro and in vivo. The upregulated IKBKBAS functioned as a competing endogenous RNA (ceRNA) via competing with IKKβ mRNA for binding miR-4741, consequently leading to upregulation and activation of IKKβ, and ultimately activation of NF-κB. The abnormally elevated IKBKBAS in LUAD was mainly resulted from the extremely decrease of miR-512-5p that targeting IKBKBAS. Furthermore, we identified a positive feedback loop between NF-κB and IKBKBAS, in which NF-κB activation induced by overexpression of IKBKBAS could promote the transcription of IKBKBAS by binding the κB sites within IKBKBAS promoter. Our studies revealed that IKBKBAS was involved in the activation of NF-κB signaling by upregulating the expression of IKKβ, which made it serve as a potential novel target for therapies to LUAD.

## Introduction

Non-small cell lung cancer (NSCLC) accounts for about 85% of all lung cancers. Among the NSCLC types, lung adenocarcinoma (LUAD) is the most common subtype [1]. LUAD is a highly heterogeneous tumor because its development is a complex process involving multi-factors. Accumulating evidence suggests that the proliferation and metastasis of LUAD cells depend on abnormal activation of multiple signaling pathways such as EGFR/MAPK, PI3/Akt, and NF-κB, etc. [2–6].

NF-κB is the key transcription factor involving in the inflammatory pathway, which makes it regarded as a key bridge connecting inflammation and tumor [7–9]. There are considerable studies indicating that NF-κB is constitutively activated in LUAD at an early stage and significantly associated with disease progression and poor prognosis of LUAD patients [9–11]. Thus, it is assumed that blockage of NF-κB will increase the efficacy of anticancer therapies. Indeed, targeted inhibition of NF-κB signaling with various approaches has been shown to augment the efficacy of chemotherapy and radiotherapy in killing LUAD cells both in vitro and in vivo [8, 12]. The canonical (classical) pathway activating NF-κB is TNF-α/IL-1β/LPS → TNFR1 → IKK complex→IκB → NF-κB (p65/p50 heterodimer). At present, the main strategies for the targeted intervention of classical NF-κB signaling pathway are [1] inhibiting the activity of NF-κB; [2] inhibiting the phosphorylation of IκBα and avoiding its ubiquitination degradation; [3] inhibiting DNA binding activity of NF-κB. Among these targets, IKKβ, one catalytic subunit of the IKK complex, is considered to be one of the most potent and selective drug targets, because phosphorylation of IκB by activated IKKβ is a prerequisite for ubiquitination degradation of IκBα and releasing of NF-κB into nuclear [6, 13, 14].

However, the underlying mechanisms for abnormal activation of NF-κB in LUAD are not completely understood. Growing evidence suggests that lncRNAs also participate in the dysregulation of NF-κB signaling. Krawczyk et al. identified lncRNA PACER promoting the transcription of COX2 gene by directly replacing p50/p50 dimer with activated p50/p65 dimer on the COX2 gene promoter [15]. Liu et al. found lncRNA NKILA binds to p65/IκBα to inhibit the phosphorylation of IκBα and p65 activation [16]. Zhang et al. found lncRNA BANCR was increased in gastric cancer and could inhibit apoptosis by inhibiting p50 expression [17]. These studies highlighted that lncRNAs play important roles in over-activation of NF-κB signaling pathway in tumors. Although IKKβ is a key link in the activation of the canonical NF-κB signaling pathway, the regulatory effect of lncRNA on its expression or activity remains rescue. In this study, we screened a novel lncRNA IKBKBAS in LUAD tissues and investigated the role of IKBKBAS in LUAD development. Ultimately, we studied the mechanism of how IKBKBAS regulates IKKβ and consequentially activates NF-κB.

## Results

### A novel lncRNA IKBKBAS is upregulated in LUAD, along with the upregulation of IKKβ and activation of NF-κB

LncRNA array was used to identify differential expression profiles of lncRNA and mRNA between 3 pairs of LUAD tissues in stage I and corresponding adjacent normal tissues. 1240 lncRNAs and 1066 mRNAs were significantly dysregulated (fold change ≥2.0, *p* < 0.05) (Figs. S1A, S1B). Interestingly, we found that the RP11-231D20.2 was upregulated with a 3.88-fold (Supplementary Table 1). RP11-231D20.2 locates in the antisense strand of IKBKB gene promoter, and the microarray data suggested that the aberrant upregulation of RP11-231D20.2 was along with the 2.21-fold increased expression of IKKβ mRNA (produced by IKBKB gene) (Supplementary Table 2) as well as the activation of NF-κB signaling pathway (Fig. S1C). Hence, we named the RP11-231D20.2 as IKBKBAS. And we identified that IKBKBAS was a 661 nucleotides transcript with poly (A) tail (Fig. S1D) that is identical to LOC101929897 (NR_125823.1, validated) in GeneBank (Fig. S1E). Typical protein-coding ORFs longer than 300 nt did not exist in IKBKBAS according to RegRNA 2.0 (http://regrna2.mbc.nctu.edu.tw/index.html) analysis (Fig. S1F).

**Fig. 1:**
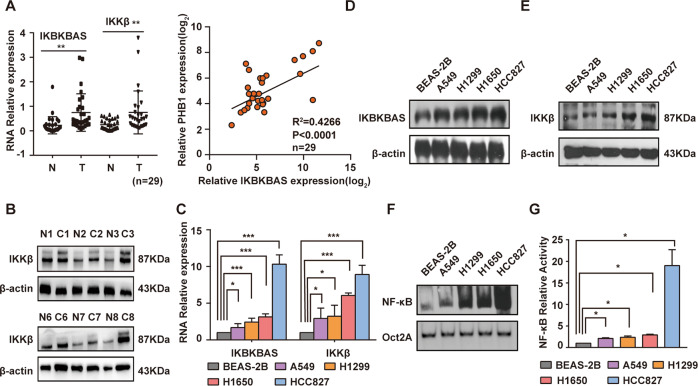
IKBKBAS is upregulated both in LUAD tissues and cells along with the upregulation of IKKβ and activation of NF-κB. **A** Validation and correlation of the IKBKBAS and IKKβ expression in LUAD specimens compared to their adjacent normal tissues by qRT-PCR analysis. **B** Partial results of western blotting on IKKβ expression in LUAD tissues compared to their adjacent normal tissues. **C** Basal expression of IKBKBAS and IKKβ in LUAD cell lines A549, H1299, H1650, HCC827, and normal cell lines BEAS-2B examined by qRT-PCR analysis. **D** Basal expression of IKBKBAS in LUAD cell lines A549, H1299, H1650, HCC827, and normal cell lineBEAS-2B examined by northern blotting. **E** Basal expression of IKKβ in LUAD cell lines A549, H1299, H1650, HCC827, and normal cell line BEAS-2B examined by western blotting. **F** Basal NF-κB activity in LUAD cell lines A549, H1299, H1650, HCC827, and normal cell line BEAS-2B showed by EMSA. **G** Luciferase reporter assay performed in LUAD cell lines A549, H1299, H1650, HCC827, and normal cell line BEAS-2B. Data show mean ± S.E.M., *n* ≥ 3, *P < 0.05, **P < 0.01, ***P < 0.01.

Then, we validated the expression levels of IKBKBAS and IKKβ in 29 paired LUAD samples with adjacent normal tissues. The results showed that IKBKBAS and IKKβ were both significantly increased in tumor group. The expression of IKBKBAS and IKKβ was positively correlated (Fig. 1A). The protein expression of IKKβ was also increased in tumor tissue (Fig. 1B). Otherwise, the IKBKBAS and IKKβ expression level was significantly increased in LUAD cell lines compared with those in normal cell lines BEAS-2B (Fig. 1C–E). Extremely high level of IKBKBAS and IKKβ was detected in HCC827, whereas relatively low level presented in A549 cells, thereby we chose these two LUAD cell lines for the following study.

Considering IKKβ is essential for NF-κB activation [18, 19], we further investigated the basal NF-κB activity in the above 4 LUAD cell lines and BEAS-2B cells. The results of EMSA also displayed that NF-κB activity was significantly higher in the 4 kinds of LUAD cell lines compared with that in BEAS-2B cells. The upregulating tendency is correspondence with the expression level of IKBKBAS and IKKβ (Fig. 1F). Consistently, dual-luciferase activity assay results also showed similar increasing tendency of NF-κB activity in A549, H1299, H1650, and HCC827 cells compared with that in normal cells BEAS-2B (Fig. 1G), conforming a positive correlation between IKBKBAS expression and NF-κB activity.

### IKBKBAS promotes LUAD proliferation and metastasis

**Fig. 2:**
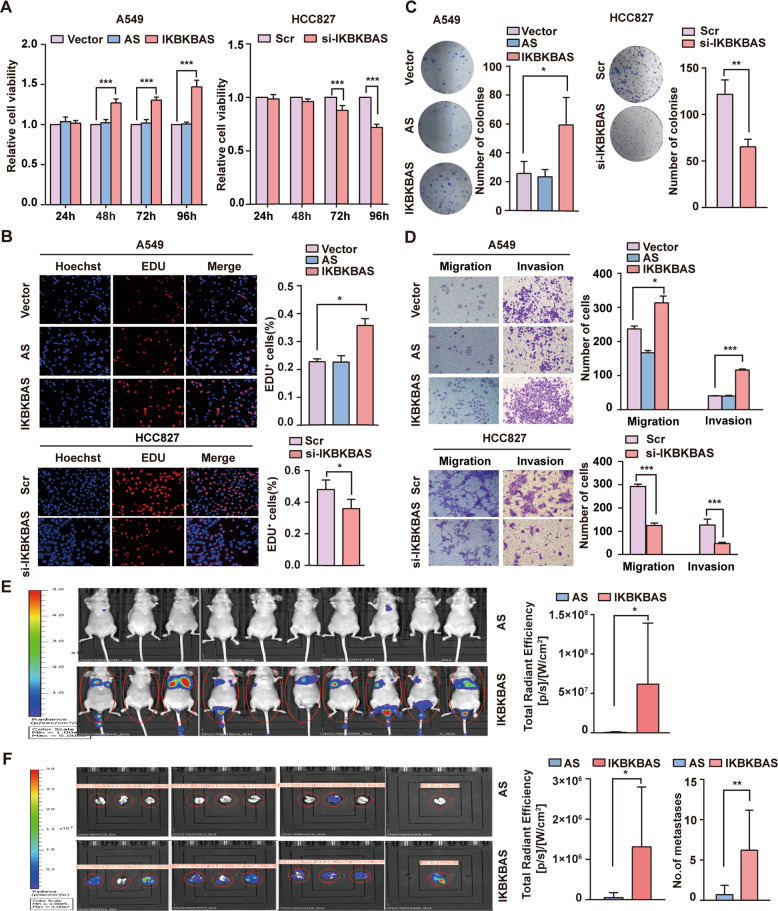
IKBKBAS promoted LUAD cells proliferation and metastasis in vitro and in vivo. Overexpression of ectopic IKBKBAS in A549 cells and knockdown of IKBKBAS in HCC827 cells were carried out for MTT assays (**A**), EdU assays (**B**), plate colony formation assays (**C**), transwell assays (**D**). **A**, **B** The effect of IKBKBAS on LUAD cell proliferation was evaluated by MTT assays and EdU assays. **C** The effect of IKBKBAS on colonizing ability was determined by plate colony formation assays. **D** The effect of IKBKBAS on invasion and migration ability was evaluated by transwell assays. **E** Luminal imaging of tumors in nude mice at 44 day following tail vein injection of IKBKBAS/IKBKBAS-AS and luciferase-expressing A549 cells were detected via a novel in vivo imaging system following luciferin injection. The red circle indicated the photon intensity in the mouse (×10^6^, mean ± SD, *n* = 10). **F** Luminal imaging for lung metastasis of A549 xenografts stably expressing IKBKBAS or IKBKBAS-AS. The red circle indicates the photon intensity in the mouse (×10^6^, mean ± SD, *n* = 10). Data show mean ± SD, *n* ≥ 3, *P < 0.05, **P < 0.01, ***P < 0.001.

The results of MTT, EdU assays, and plate colony formation assay indicated that ectopic IKBKBAS expression increased proliferation of A549 cells. Conversely, knockdown of IKBKBAS reduced the proliferation of HCC827 cells (Fig. 2A–C). Furthermore, transwell assays demonstrated that IKBKBAS overexpression promoted invasion and metastasis capability of A549 cells, whereas IKBKBAS knockdown inhibited invasion and metastasis capability of HCC827 cells (Fig. 2D). The similar results were shown on H1299 cells (Fig. S2). In addition, tail vein metastasis showed that IKBKBAS could promote lung cancer metastasis (Fig. 2E, F). Collectively, these in vitro and in vivo results highlighted the functional significance of IKBKBAS-promoted malignant progression of LUAD.

### IKBKBAS is a positive regulator of NF-κB signaling pathway

**Fig. 3:**
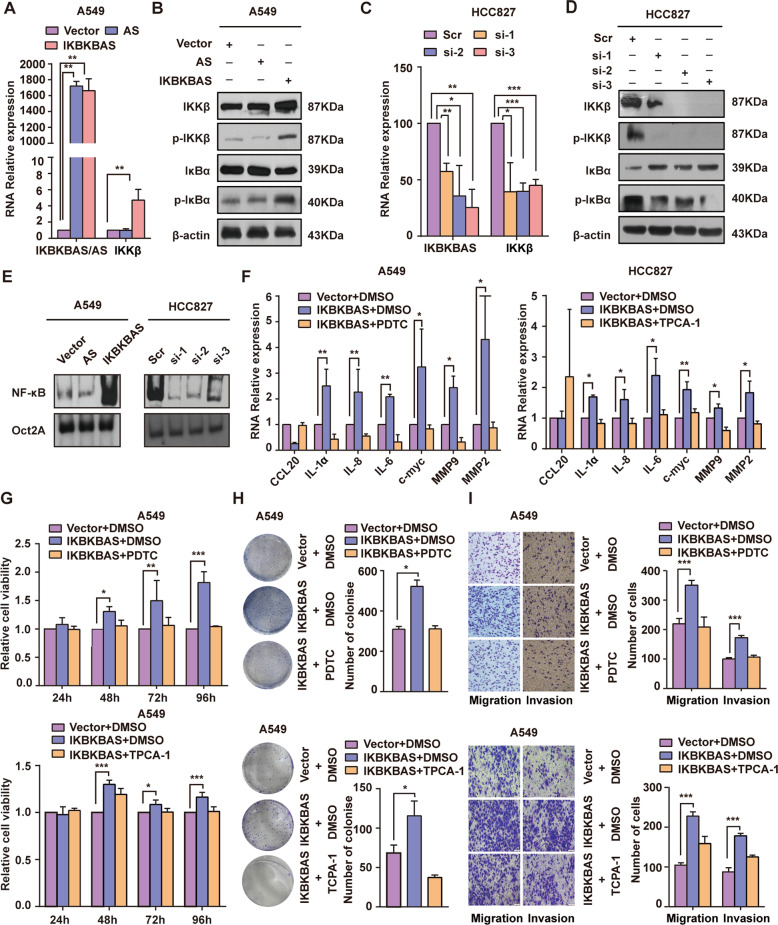
IKBKBAS is a positive regulator of NF-κB signaling pathway. **A** The relative expression of IKKβ in A549 cells with ectopic expression of IKBKBAS were assayed by qRT-PCR. **B** Western blot analysis for the expression of IKKβ, p-IKKβ, IκBα, and p-IκBα in A549 cells with ectopic expression of IKBKBAS. **C** The relative expression of IKKβ in HCC827 cells with knockdown of IKBKBAS assayed by qRT-PCR. **D** Western blot analysis for the expression of IKKβ, p-IKKβ, IκBα, and p-IκBα in HCC827 cells with knockdown of IKBKBAS. **E** Activity of NF-κB in A549 cells with ectopic expression of IKBKBAS and activity of NF-κB in HCC827 cells with knockdown of IKBKBAS were detected by EMSA. **F** The relative expression of target genes of NF-κB were assayed by qRT-PCR in A549 cells, including IL-1α/6/8, MMP9/2, c-myc, CCL20. The A549 cells were treated with ectopic expression of IKBKBAS, and with treatment of NF-κB inhibitor PDTC and IKKβ inhibitor TPCA-1, respectively. **G** MTT assay showing the effect of IKBKBAS on cell proliferation in A549 cells with PDTC or TPCA-1 treatment. **H** Plate colony formation assays showing the effect of IKBKBAS on colonizing ability in A549 cells with PDTC or TPCA-1 treatment. **I** Transwell assays exhibited the effect of IKBKBAS on invasion and migration ability in A549 cells with PDTC or TPCA-1 treatment.Data show mean ± SD, *n* ≥ 3, *P < 0.05, **P < 0.01, ***P < 0.001. si-1: si-288; si-2: si-449; si3: si-288+si-449.

As shown in Fig. 3A–D, ectopic expression of IKBKBAS in A549 cells significantly increased the IKKβ expression at both mRNA and protein level as well as phosphorylation of IKKβ (p-IKKβ) and IκBα (p-IκBα). On the contrary, IKBKBAS knockdown in HCC827 cells led to decrease in the level of IKKβ expression as well as IKKβ and IκBα phosphorylation. Meanwhile, the results of EMSA also showed ectopic expression of IKBKBAS in A549 cells significantly enhanced NF-κB activity, whereas IKBKBAS knockdown in HCC827 cells led to reduced NF-κB activity (Fig. 3E). The above experiments were also performed in H1299 cells and presented similar tendency (Fig. S3). The functional positive regulator of IKBKBAS on NF-κB signaling was further illustrated by rescue experiments. IKBKBAS overexpression in A549 cells resulted in correspondingly upregulation or downregulation of NF-κB target genes, including IL-1α/6/8, MMP9/2, c-myc, CCL20, respectively. Expectedly, all these regulation effects were attenuated by treatment with NF-κB inhibitor PDTC or IKKβ inhibitor TPCA-1 (Fig. 3F). Moreover, as shown in Fig. 3G–I, enhanced proliferation, invasion, and metastasis abilities of A549 cells caused by IKBKBAS overexpression were attenuated by PDTC or TPCA-1 treatment. Collectively, the results above suggested that the carcinogenesis role of IKBKBAS in LUAD cells was mediated, at least in part, by activating NF-κB signaling.

### Cytoplasmic IKBKBAS upregulates IKKβ at post-transcriptional level

**Fig. 4:**
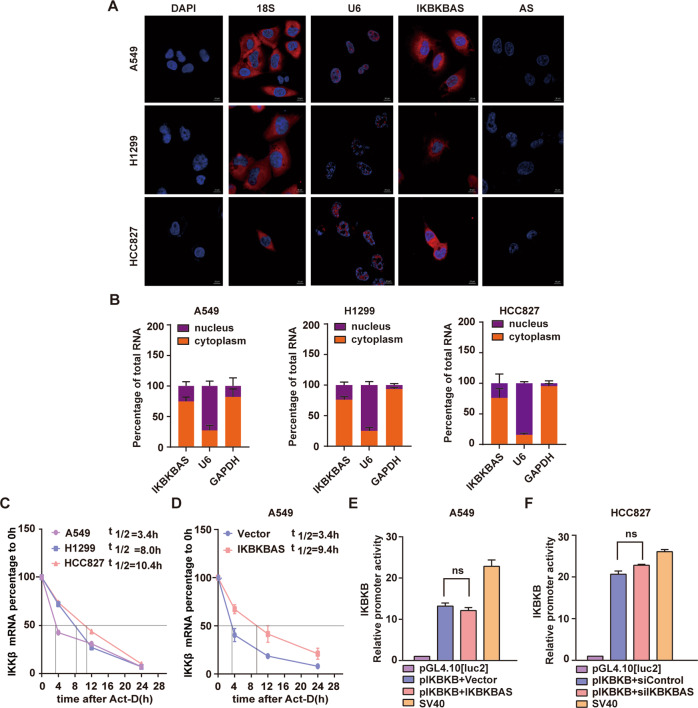
Cytoplasmic IKBKBAS upregulates IKKβ at post-transcriptional level. **A** Confocal FISH images showing cytoplasmic localization of IKBKBAS in A549, H1299, and HCC827 cells, respectively. The nuclei were stained with DAPI, U6 snRNA, and 18S rRNA was used as a nuclei and cytoplasmic marker, respectively. IKBKBAS-AS were used as negative control. Scale bar: 10 μm. **B** Cell nuclear/cytoplasmic fractionation and qRT-PCR assay showing the cellular distribution of IKBKBAS in A549, H1299, and HCC827 cells, respectively. U6 and GAPDH were used as separation quality standards and endogenous controls. **C** The kinetics of IKKβ mRNA in A549, H1299, and HCC827 cells treated with Act-D were assayed by qRT-PCR, respectively. **D** The kinetics of IKKβ mRNA in A549 cells with ectopic IKBKBAS expression and Act-D treatment were assayed by qRT-PCR. **E** Luciferase reporter assay showing the effect of IKBKBAS on promoter activity of IKBKB gene in A549 cells with ectopic expression of IKBKBAS. **F** Luciferase reporter assay showing the effect of IKBKBAS on promoter activity of IKBKB gene in HCC827 cells with knockdown of IKBKBAS expression.

Given the critical role of IKBKBAS in NF-κB signaling activation, we then sought to explore the underlying molecular mechanism by which IKBKBAS upregulated IKKβ. We first used confocal microscopy for RNA FISH to verify the subcellular location of IKBKBAS and found it located primarily in the cytoplasm (Fig. 4A). The cytoplasmic IKBKBAS was further confirmed by nuclear/cytoplasm fractionation (Fig. 4B and Fig. S4). To investigate the underlying mechanism of IKBKBAS upregulating IKKβ, we then examined the half-life of IKKβ mRNA in LUAD cells. The results indicated that half-life of IKKβ mRNA in A549, H1299, and HCC827 was about 3.4, 8.0, 10.4 h (Fig. 4C), respectively, presenting positive correlation with IKKβ expression level of each cell. Moreover, ectopic expression of IKBKBAS extended half-life of IKKβ mRNA from 3.4 to 9.4 h in A549 cells (Fig. 4D). Furthermore, the results of dual-luciferase activity assay indicated that neither IKBKBAS overexpression nor IKBKBAS silencing exerted influence on promoter activity of IKBKB gene (IKKβ) (Fig. 4E, F), suggesting that IKBKBAS upregulates IKKβ via stabilizing the IKKβ mRNA. Based on the results above, we surmised that IKBKBAS may be a ceRNA for IKKβ mRNA.

### IKBKBAS competes for binding of miR-4741 with IKKβ mRNA

**Fig. 5:**
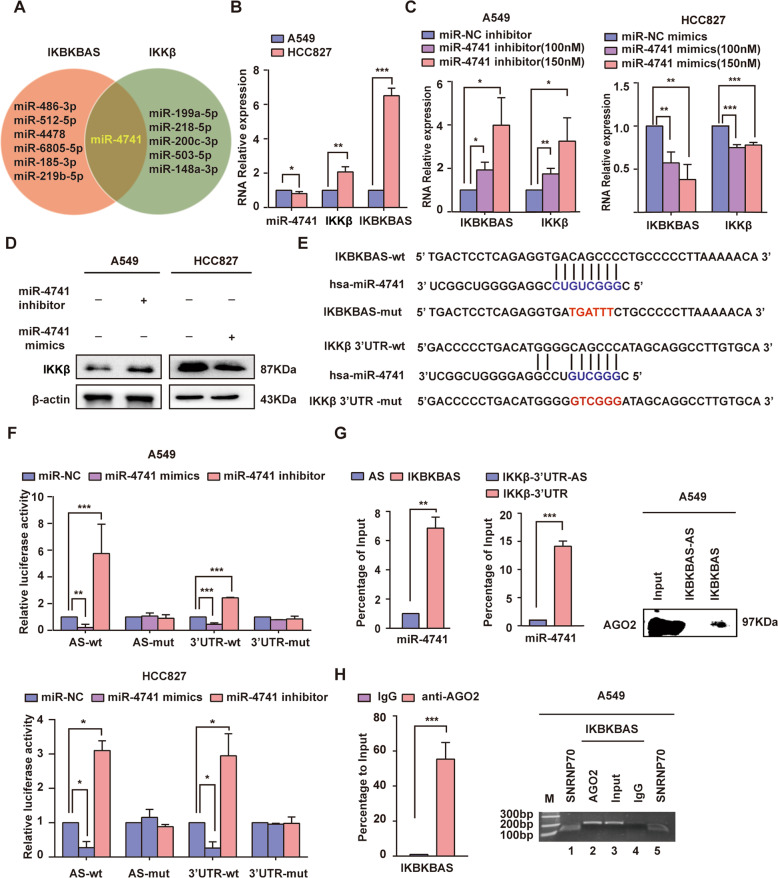
IKBKBAS competes for binding of miR-4741 with IKKβ mRNA. **A** The common miRNA that targeted to both IKBKBAS and IKKβ mRNA predicted by RegRNA 2.0, Targetscan Human 7.1, and miRDB. **B** The relative expression of miR-4741, IKBKBAS, and IKKβ mRNA in A549 and HCC827 cells were assayed by qRT-PCR. **C** Validation of the effect of miR-4741 inhibitor or mimics on expression of IKKβ in A549 and HCC827 cells by qRT-PCR analysis. **D** The effect of miR-4741 inhibitor or mimics on expression of IKKβ in A549 and HCC827 cells was assayed by western blot. **E** Schematic representation of the predicted MREs for miR-4741 on IKBKBAS or IKKβ 3′UTR, and the site mutagenesis design for the luciferase reporter. **F** Luciferase reporter assay confirmed the interaction between miR-4741 and IKBKBAS or IKKβ 3′UTR in A549 and HCC827 cells. A549 and HCC827 cells were co-transfected with miR-4741 mimic or miR-4741 inhibitor and pMIR-IKBKBAS-wt/mut or pMIR-IKKβ-3′UTR-wt/mut plasmids. miR-NC mimics or miR-NC inhibitor were also transfected as control. Transfected cells were harvested after 48 h and subjected to luciferase activity assay. **G** The association among IKBKBAS, miR-4741, IKKβ 3′UTR, and AGO2 was ascertained by RNA pull-down assay using A549 cell lysates. Left: Detection of miR-4741 by qRT-PCR in the sample pulled down using biotinylated IKBKBAS and IKBKBAS-AS (negative control) probes; middle: Detection of miR-4741 by qRT-PCR in the sample pulled down using biotinylated IKKβ 3′UTR and IKKβ 3′UTR-AS (negative control) probes; right: Detection of AGO2 by western blot in the sample pulled down using biotinylated IKBKBAS and IKBKBAS-AS (negative control) probes. **H** The association between IKBKBAS and Ago2 was ascertained by RIP assay using A549 cell lysates with an AGO2 antibody. The anti-SNRNP70 and IgG were used as positive and negative control, respectively. Left: qRT-PCR result; right: RT-PCR result (lane M: marker; lanes 2–4: IKBKBAS fragment, 208 bp; lane 1, 5: U1 snRNA,118 bp). Data show mean ± S.E.M., *n* ≥ 3, *P < 0.05, **P < 0.01, ***P < 0.001.

To screen miRNAs targeting both IKBKBAS and IKKβ mRNA, we searched potential miRNA response elements presented in IKBKBAS and IKKβ 3′UTR using RegRNA2.0, TargetScanHuman 7.1 (http://www.targetscan.org/vert_71/), and miRBD (http://mirdb.org/), respectively. The overlap of screening results revealed that miR-4741 maybe could target both IKBKBAS and IKKβ mRNA (Fig. 5A). Thereafter, we performed qRT-PCR to compare the expression levels of miR-4741, IKBKBAS, and IKKβ between A549 cells and HCC827 cells. Compared to the significant high expression level of IKBKBAS and IKKβ in HCC827, miR-4741 level was downregulated expression between these two cell lines (Fig. 5B). This result suggested that increased IKBKBAS sponged more miR-4741 and thus resulted in upregulation of IKKβ. Further, the results of qRT-PCR and western blot indicated that miR-4741 silencing in A549 cells significantly increased the expression of IKBKBAS and IKKβ, whereas overexpression of miR-4741 in HCC827 cells decreased the expression of them (Fig. 5C, D). Meanwhile, the reporter gene assays displayed that overexpression of miR-4741 significantly repressed the luciferase activity of the luciferase fused to IKBKBAS (luc-IKBKBAS) or IKKβ-3′ UTR (luc-IKKβ-3′ UTR) in both A549 and HCC827 cells. However, miR-4741 silencing resulted in upregulation of luciferase activity, suggesting the binding of miR-4741 to both IKBKBAS and IKKβ-3′ UTR (Fig. 5E, F). Moreover, lncRNA pull-down assays and qRT-PCR were conducted to verify the direct interaction between miR-4741 and IKBKBAS or between miR-4741 and IKKβ 3′ UTR. In parallel, the RNA–protein complexes were subjected to western blot with Anti-Ago2. The result confirmed that the IKBKBAS was involved in the formation of RNA-induced silencing complex (RISC) (Fig. 5G). To further confirm the involvement of IKBKBAS in RISC, we performed RIP, and found robust enrichment of IKBKBAS in the anti-Ago2 antibody-interacting RNA fraction compared to IgG control (Fig. 5H).

### Downregulation of miR-512-5p targeting IKBKBAS in LUAD cells results in upregulation of IKBKBAS expression

**Fig. 6:**
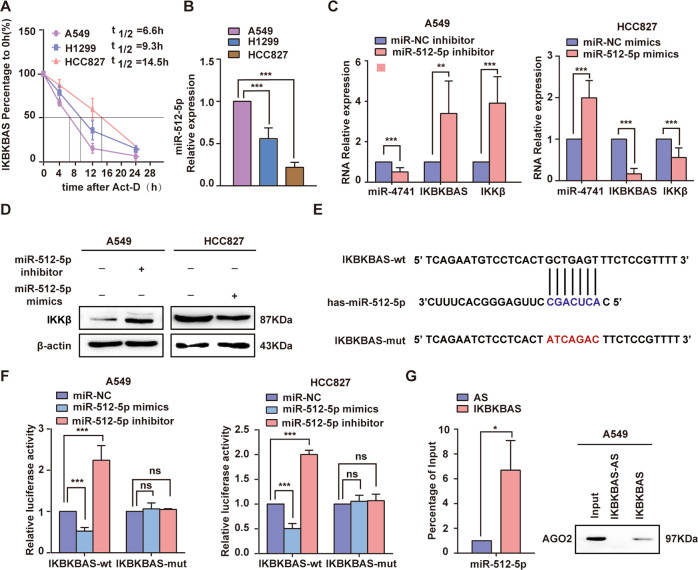
miR-512-5p is extremely downregulated in LUAD cells and targets IKBKBAS. **A** Half-life of IKBKBAS in A549, H1299, and HCC827 cells detected by qRT-PCR. **B** Basal expression of miR-512-5p in A549, H1299, or HCC827 cells detected by qRT-PCR. **C** The effect of miR-512-5p inhibitor or mimics on expression of miR-4741, IKBKBAS, or IKKβ mRNA in A549 (left) and HCC827 (right) cells was assayed by qRT-PCR. **D** The effect of miR-4741 inhibitor or mimics on expression of IKKβ in A549 and HCC827 cells were assayed by western blot. **E** Schematic representation of the predicted binding sites for miR-512-5p on IKBKBAS and IKKβ 3′UTR, and the site mutagenesis design for the luciferase reporter assay. **F** Luciferase reporter assay confirmed the interaction between miR-512-5p and IKBKBAS in A549 and HCC827 cells. A549 and HCC827 cells were co-transfected with miR-512-5p mimic or miR-512-5p inhibitor and pMIR-IKBKBAS-wt/mut plasmids.miR-NC mimics or miR-NC inhibitor were also transfected as control. Transfected cells were harvested after 48 h and subjected to luciferase activity assay. **G** The association among IKBKBAS, miR-512-5p, and AGO2 was ascertained by RNA pull-down assay using A549 cell lysates. Left: Detection of miR-512-5p by qRT-PCR in the sample pulled down using biotinylated IKBKBAS and IKBKBAS-AS (negative control) probes; right: Detection of AGO2 by western blot in the sample pulled down using biotinylated IKBKBAS and IKBKBAS-AS (negative control) probes. Data show mean ± S.E.M., *n* ≥ 3, *P < 0.05, **P < 0.01, ***P < 0.001.

To explore the causes of the high expression of IKBKBAS in LUAD cells, we first measured the half-life of IKBKBAS in A549, H1299, and HCC827 cells, respectively. The half-life of IKBKBAS is much longer in HCC827 cells (14.5 h) than that in A549 cells (6.6 h) upon transcriptional inhibition by Act-D, while half-life of IKBKBAS in H1299 (9.3 h) is between that in HCC827 and A549 (Fig. 6A). This result consisted with the expression level of IKBKBAS and basal activity of NF-κB among the three cell lines, suggesting that the abnormal upregulation of IKBKBAS may take place at the post-transcriptional level. Thereby, we detected miRNAs only targeting to IKBKBAS but not IKKβ in A549 cells and HCC827 cells. qRT-PCR analysis indicated that the expression of miR-512-5p and miR-486-3p was dramatically decreased in HCC827 cells compared to that in A549 cells (Fig. S5A). It is reported that miR-512-5p is a tumor suppressor and significantly downregulated in NSCLC patient tumor samples compared to that in paired normal lung tissues [20, 21]. Also, we found that miR-512-5p was much more highly expressed in A549 than that in HCC827 cells (Fig. 6B). Then gain/loss of function analysis showed that knockdown of miR-512-5p downregulated the miR-4741 in A549 cells, while upregulating IKBKBAS and IKKβ expression in A549 and H1299 cells. In parallel, overexpression of miR-512-5p in HCC827 cells upregulated the miR-4741, but decreased IKBKBAS and IKKβ (Fig. 6C, D; Fig. S5C, D). However, the knockdown or overexpression of miR-486-3p almost had little effect on expression of IKBKBAS, or IKKβ (Fig. S5B). To verify whether the upregulation of IKBKBAS in LUAD cells resulted from the significant downregulation of miR-512-5p, we performed the dual-luciferase activity analysis and miRNApull-down assays. Both the results displayed the direct interaction between miR-512-5p and IKBKBAS (Fig. 6E–G).

### Reciprocal positive regulation is existed between IKBKBAS and NF-κB

**Fig. 7:**
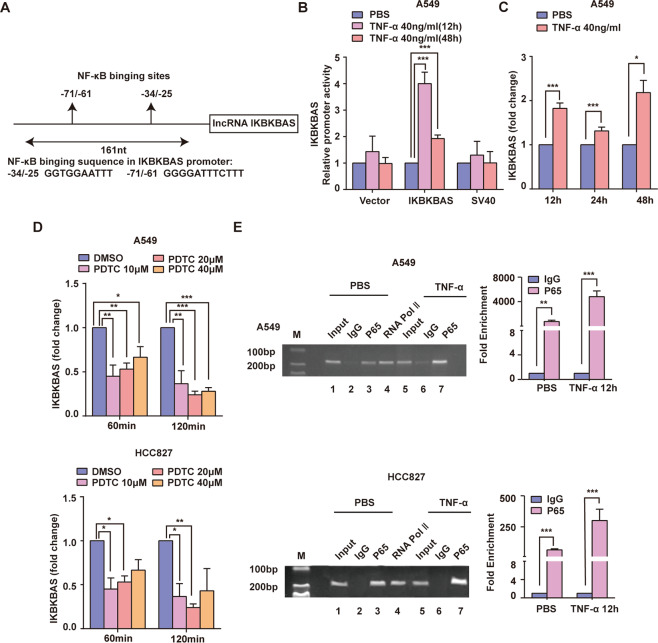
Reciprocal positive regulation between IKBKBAS and NF-κB. **A** Schematic representation of the predicted binding sites for NF-κB on IKBKBAS promoter for ChIP assay. **B** Luciferase reporter assay showing the relative promoter activity of IKBKBAS in A549 cells treated with TNFα 40 ng/ml for 12, 24 h, respectively. **C** The expression of IKBKBAS in A549 cells treated with TNFα 40 ng/ml for 12, 24, 48 h was detected by qRT-PCR, respectively. **D** Expression of IKBKBAS detected in A549 cells and HCC827 cells with treatment of PDTC (10, 20, 40 μM) for 60 min, 120 min by qRT-PCR. **E** The interaction between p65 and two κB sites on IKBKBAS promoter were assayed by ChIP experiments. The anti-p65 or IgG antibody (negative control) or anti-RNA pol II (positive control) in A549 cells and HCC827 cells treated with TNFα (40 ng/ml) for 12 h were performed. Left: the RT-PCR results (lane M: marker; lanes 1–3 & 5–7: IKBKBAS promoter DNA fragment containing 2 κB sites, 161 bp; lane 4: GAPDH promoter DNA fragment: 166 bp); right: qRT-PCR results. Y axis represents the % input of the promoter fragments captured by the two different antibodies. Data show mean ± S.E.M., *n* ≥ 3, *P < 0.05, **P < 0.01, ***P < 0.001.

By analyzing the sequence across IKBKBAS promoter using TRANSFEC4.0 (http://gene-regulation.com/pub/databases.html), two potential κB enhancer elements were found (Fig. 7A). Furthermore, results of dual-luciferase activity assay displayed that TNF-α treatment significantly enhanced the promoter activity of IKBKBAS in A549 cells (Fig. 7B). Hence, we suspected that NF-κB also is implicated in the regulation of IKBKBAS expression. To prove this hypothesis, we detected IKBKBAS expression level after activating NF-κB by TNF-α or inhibiting NF-κB activity by PDTC. qRT-PCR analysis showed that the expression of IKBKBAS was increased when cells were treated with TNF-α while decreased when treated with PDTC (Fig. 7C, D). To confirm these results, we designed primers to amplify 161 bp segments spanning both predicted κB binding sites and performed ChIP assays to examine whether NF-κB can directly bind to the two sites on IKBKBAS promoter. The results showed that p65 was recruited to κB sites with or without TNF-α treatment, whereas occupancy of the κB sites was markedly increased upon TNFα stimulation for 12 h (Fig. 7E). These observations suggest that IKBKBAS is a potent NF-κB transcriptional target, and there is a positive feedback loop existing between IKBKBAS expression and NF-κB activating.

## Discussion

Deregulated activation of NF-κB is widespread in human cancers include LUAD [22]. Considering that there are few mutations affecting the functions of NF-κB and its regulatory IκB subunits, the abnormally high activity of NF-κB is largely due to regulatory network dysfunction of its own signaling pathway [23, 24]. The regulation of NF-κB signaling pathway by miRNAs and proteins has been extensively studied [25, 26]. During last 5 years, increasing evidence indicated that NF-κB signaling was also regulated directly or indirectly by lncRNAs, including NKILA, HOTAIR, MALAT1, BANCR, GAS5, HOXA-AS2, FAM3D-AS1, TP73-AS1, and lnc-DILC, etc. [17, 16, 27–34,]. Therefore, NF-κB signaling regulation is based on a complex network consisted of numerous proteins and non-coding RNAs via intermolecular interaction. It is a reasonable to infer that there may be other lncRNAs involved in this regulation network for NF-κB signaling.

Our results showed that a novel lncRNA, IKBKBAS, was upregulated along with IKKβ upregulation as well as NF-κB signaling enhancing in LUAD. IKBKBAS locates in the antisense strand of IKBKB promoter region, which belongs to antisense lncRNAs subclass as well as so-called natural antisense RNA transcripts (NAT) [35, 36]. NATs carry out a wide variety of biological roles that are important for the normal functioning of living cells mainly by affecting the expression of its sense partner, and their dysfunctions can lead to the development of diseases including cancer [36–39]. Therefore, we inferred that IKBKBAS positively regulates the expression of IKKβ and thus plays an important regulatory role in NF-κB signaling. To verify this assumption, we performed gain-of-function and loss-of-function analysis in LUAD cells. The results of a following serious of functional assays indicated that IKBKBAS promotes LUAD cells proliferation, invasion and metastasis in vitro. Furthermore, tail vein metastasis assay with nude mice demonstrated that IKBKBAS increases metastasis activity of LUAD cell in vivo. Collectively, IKBKBAS may function as an oncogenic lncRNA in LUAD. The oncogenic function of IKBKBAS is mediated by activating NF-κB signaling pathway via upregulating IKKβ.

The majority of NATs modulate expression of their sense partners by diverse transcriptional and post-transcriptional regulatory mechanisms [38]. IKBKBAS is possible a cytoplasmic lncRNA because it has a poly(A) tail which is essential for nuclear export and cytoplasmic function [40–42]. This was demonstrated by RNA FISH, subcellular fractionation assay, and qRT-PCR. Further, we observed that the half-life of IKKβ mRNA in A549, H1299, and HCC827 cells increased, consistent with their increased IKBKBAS expression level. Moreover, overexpressed IKBKBAS in A549 cells can extend the half-life of IKKβ mRNA, but has no significant effect on promoter activity of IKBKB gene, suggesting that it upregulates IKKβ at the post-transcriptional level. There may be two possible molecular mechanisms: IKBKBAS either work through direct RNA–RNA interactions to form duplex RNA masking MREs on the target RNA, or function as a miRNA sponge [38, 43]. Since the IKBKBAS gene locates in the antisense strand of promoter region of IKBKB, it has no complementary overlap with the mature IKKβ mRNA. We inferred that IKBKBAS functions as miRNA sponge for IKKβ mRNA. Through bioinformatics analysis and gain or loss of function assays, we found that the abnormally overexpressed IKBKBAS, as a molecular sponge, enhances the stability of IKKβ mRNA by competing with IKKβ to bind to miR-4741, resulting in enrichment of IKKβ and consequently activation of IKKβ via trans auto-phosphorylation [44] in LUAD, finally activating NF-κB.

Subsequently, we explored the mechanism underlying abnormal upregulation of IKBKBAS in LUAD. We found that miR-512-5p was significantly decreased in both HCC827 cells and H1299 cells compared to A549 cells, consistent with observation by other researchers [20, 45], but negatively correlated with the expression level of IKBKBAS. In addition, all the data suggest miR-512-5p downregulates IKBKBAS, which further increase the expression of IKKβ by competitively binding to the miR-4741. The miR-512-5p is a known tumor suppressor miRNA and is observed downregulated in a variety of human carcinomas including LUAD [20, 21, 45–49]. For instance, Chu et al. reported that miR-512-5p promoted apoptosis, and inhibited glycolysis in LUAD cells by targeting p21 [20]. And Wang et al. also found miR-512-5p attenuating A549 cells migration by targeting beta-catenin [45]. Taken together with our results, the inhibition of migration by miR-512-5p might be at least partly mediated through indirectly activating NF-κB signaling by targeting IKBKBAS. Several studies reported that downregulation of miR-512-5p in cancers was most largely due to hypermethylation of CpG islands in promoter region of miR-512 [21, 48, 50].

The data accumulated so far implicated that, regulators like protein, miRNA, and lncRNA for NF-κB signaling can also be induced by NF-κB inducing stimuli and thus are involved in the feedback regulation [51]. Similarly, we found that IKBKBAS promoter also contains two κB elements by software scanning. As expected, TNFα stimulation can increase the transcriptional activity of IKBKBAS, while treatment with the p65 inhibitor PDTC, has an opposite effect. Furthermore, ChIP assay demonstrated that p65 directly bound the two κB sites within promoter of IKBKBAS. Thus, there was a positive feedback loop between activating NF-κB and IKBKBAS expression.

In conclusion, we identified a novel lncRNA IKBKBAS localized in the opposite DNA strand in the promoter of IKBKB. IKBKBAS functions as ceRNA by competing with IKKβ mRNA for sponging miR-4741, leading to upregulation and activation of IKKβ, and finally resulting in the activation of NF-κB signaling pathway. The abnormal upregulation of IKBKBAS in LUAD cells is due to the repressed expression of miR-512-5p caused by its promoter methylation. Furthermore, the activated NF-κB binds to the κB sites within promoter of IKBKBAS resulting in transcriptional activating of IKBKBAS, forming a positive feedback loop between IKBKBAS and NF-κB. These findings verified the importance of IKBKBAS in promoting LUAD development and progression, and IKBKBAS may be a promising molecular target for the treatment of LUAD.

## Materials and methods

### Reagents

Recombinant human TNF-α (210-TA/CF) was purchased from R&D Systems (Minneapolis, MN, USA). Ammonium pyrrolidinedithiocarbamate (PDTC) were purchased from Abcam (Cambridge, UK). TPCA-1 (GW683965) was purchased from Selleck (Shanghai, China). Lipofectamine3000, Bio-16-UTP, Streptavidin agarose beads, and TRIzol reagent were purchased from Thermo Fisher Scientific Life Sciences (Waltham, MA, USA). Actinomycin D (Act D) was purchased from Sigma-Aldrich (St Louis, MO, USA). Protease Inhibitor Cocktail tablets were purchased from Roche (Basel, Switzerland). Phosphatase inhibitor was purchased from Solarbio Science & Technology Co. (Beijing, China). The fluorescence-labeled probes for lncRNA fluorescence in situ hybridization (FISH) assays, miRNA mimics/inhibitors and miRNA primers were obtained from RiboBio Co. Ltd (Guangzhou, China).

### Tissue specimens

A total of 29 pairs of fresh LUAD and adjacent normal tissue specimens were obtained from Qilu Hospital of Shandong University (Jinan, China). All patients were treatment naïve before surgery. Our study was approved by the Institute’s Research Ethics Committee of Qilu Hospital Shandong University (KYLL-2017(KS)-118) and conducted in accordance with ethical guidelines of the World Medical Association Declaration of Helsinki. Informed consent was written by all patients prior to this study.

### Plasmid construction and establishment of stable cell lines

The IKBKBAS expression plasmid and its control, pcDNA3.1(+)-IKBKBAS and pcDNA3.1(+)-IKBKBAS-AS (antisense chain of IKBKBAS), were produced by inserting IKBKBAS cDNA into pcDNA3.1(+) plasmid bidirectionally as well as pcDNA3.1(+)-IKKβ 3′UTR and pcDNA3.1(+)‑IKKβ 3′UTR-AS. The luciferase reporter gene plasmids, pGL4.10-pIKBKB and pGL4.10-pIKBKBAS, were constructed by amplifying IKBKB promoter DNA (1155 bp; −750/+405) and IKBKBAS promoter DNA (440 bp; −418/+22) using the Prime STAR HS DNA Polymerase (Takara, Tokyo, Japan), then inserting into pGL4.10 [luc2] Vector, respectively. In addition, fragments containing the binding sites (wild type or mutant) for miR-4741 in IKBKBAS and IKKβ 3′UTR, or miR-512-5p in IKBKBAS were synthesized by Biosune Biotechnology CO., Ltd (Shanghai, China) and subcloned into the pMIR-Report Luciferase Vector to generate pMIR–IKBKBAS wt/mut (4741), pMIR–IKBKBAS wt/mut (512), and pMIR-IKKβ 3′UTR wt/mut, respectively.

### Cell culture and transfection

Human lung normal epithelial cell line BEAS-2B, LUAD cell lines A549, HCC827, H1299, and NCI-H1650 were provided by Cell Bank (CAS). All the cells were cultured with appropriate media and supplemented with 10% fetal bovine serum (BI, Israel). All cells were maintained in a humidified 5% CO_2_ atmosphere at 37 °C. Cells were transiently transfected with miRNA mimics/inhibitor, siRNA (GenePharma, Shanghai, China), plasmids and the corresponding control using Lipofectamine3000 following the manufacturer’s recommendations. Transfection efficiency was confirmed by qRT-PCR as well as western blotting assays. Sequences or ID numbers of miRNA mimics/inhibitor and siRNAs used in this study were given in Supplementary Table 3.

### RNA extraction and quantitative real-time PCR (qRT-PCR)

RNA extraction and qRT-PCR were performed according to the manufacturer’s instructions. TRIzol reagent was applied for total RNA extraction. First-strand cDNA was synthesized using RevertAid First Strand cDNA Synthesis Kit (Thermo Fisher Scientific). qRT-PCR analysis was performed using SYBR^®^Premix Ex Taq™ (Takara) with Bio-Rad CFX96 Touch Real-time PCR Detection System (Bio-Rad, Berkeley, CA, USA). For detecting miRNA expression, reverse transcription and qPCR assays were performed using Bulge-Loop miRNA qRT-PCR Starter Kit with Bulge-Loop^TM^ miRNA Primer Set (RiboBio) according to the manufacturer’s instructions. β-actin and U6 were used as an endogenous control for mRNA and miRNA, respectively. Sequences of primers used were given in Supplementary Table 3.

### Western blot analysis and antibodies

Tissues or cells were lysed in RIPA buffer (Thermo Fisher Scientific) supplemented with protease and phosphatase inhibitors. The detailed steps are similar to a previous study [52]. Antibodies used in this study include anti-IKKβ, anti-P-IKKβ, anti-IκBα, anti-P-IκBα, anti-GAPDH, anti-β-actin, anti-H3, and others. More information about antibodies were listed in Supplementary Table 3.

### Northern blotting

Total RNA was isolated from BEAS-2B and LUAD cells using RNeasy® Mini Handbook (Qiagen, Hilden, Germany) according to the manufacturer’s instructions. 3′-end-DIG-labeled IKBKBAS and β-actin probes were synthesized by Sangon Biotech (Shanghai, China). The detailed steps are similar to a previous study [53]. The sequences of DIG-labeled IKBKBAS and β-actin probes are listed in Supplementary Table 3.

### Dual-luciferase assay

Dual-luciferase assay was performed as previously described [54]. Briefly, cells transfected with indicated plasmids or miRNA mimics/inhibitor were harvested and subjected to luciferase reporter assay using the Dual Luciferase Assay Reporter System according to the manufacturer’s instructions (Promega, Madison, WI, USA).

### Electrophoretic mobility shift assays (EMSA)

EMSA was performed using the DIG Gel Shift Kit, 2nd Generation (Sigma-Aldrich), and according to the methods described in our previous study [55]. Nuclear protein was extracted from BEAS-2B and LUAD cells by Nuclear and Cytoplasmic Protein Extraction Kit (Beyotime). NF-κB probes was synthesized by Sangon Biotech. The sequences of EMSA DNA probes are listed in Supplementary Table 3.

### MTT assays

Cells were transfected for 24 h and then were seeded in a 96-well plate in a density of 5 × 10^3^ cells/well. 24, 48, 72, and 96 h later, the cells were co-incubated with 10 μl 5 mg/ml MTT solution (Sigma-Aldrich) for 4 h. Thereafter, 100 μl DMSO was added to dissolve the formazan crystal after removing supernatant. The cell viability was then detected by reading the A_570_ value normalizing to non-treated cells. All assays were performed in triplicate.

### Transwell migration and invasion assays

Cells were seeded into Transwell migration chambers with a pore size of 8 µm (Corning, New York, NY, USA). The upper chamber was either left uncoated for the migration assay or precoated with 50 µl of 1:8 diluted Matrigel (Corning) for the invasion assay. All these experiments were performed in triplicate.

### Plate colony formation assays

Plate colony formation assays were performed in six-well culture plates. Treated cells were seeded at a density of 4 × 10^2^/well. The cells were incubated at 37 °C for 2 weeks and then washed three times with PBS before fixing and staining with Giemsa Stain Solution. The number of colonies containing ≥50 cells was counted using a microscope.

### EdU assays

Cell proliferation was determined using the Cell Light™ EdU Apollo567 In Vitro Kit (RiboBio) following the manufacturer’s instructions.

### Tail vein metastasis assays

Male BALB/c nude mice (5 weeks, 18–20 g) were obtained from Shanghai Lingchang Biological Technology Co., Ltd (Shanghai, China). Twenty mice were randomly assigned to two groups (10 mice/group). To generate organ metastases, a total of 2 × 10^6^ A549^luc-IKBKBAS^ or A549^luc-IKBKNAS-AS^ cells suspended in 100 μl serum-free medium were injected intravenously into mice through tail vein. After intraperitoneal injection of D-Luciferin (15 mg/mL) at 8, 14, 23, 30, 37, and 44 days, metastasis effect of IKBKBAS on cells was assessed by dynamically detecting luciferase-mediated bioluminescence signals using a small animal in vivo imaging system (Perkin Elmer, Waltham, MA, USA). At 44 day, mice were sacrificed, and the lungs were isolated for examination of bioluminescence signals.

### RNA stability assays

LUAD cells were plated into 35 mm dish, treated with 5 μg/ml Act D for 0–24 h, respectively. The total RNAs were then extracted by TRIzol at indicated time points and analyzed by qRT-PCR.

### lncRNA fluorescence in situ hybridization (FISH)

The RNA FISH assay was performed in cells with the FISH Kit (RiboBio) according to the manufacturer’s protocol. All detailed information for probes were listed in Supplementary Table 3.

### Subcellular fractionation

The separation of the nuclear and cytosolic fractions was performed with a PARIS™ Kit (Thermo Fisher Scientific) following the manufacturer’s instructions. U6 were used as endogenous controls for the nucleus, whereas GAPDH for the cytoplasm. The sequence of primers used for PCR are listed in Supplementary Table 3. Cytoplasmic GAPDH and nuclear H3 were also detected by western blot as quality control.

### RNA pull‑down assay

pcDNA3.1(+)‑IKBKBAS, pcDNA3.1(+)‑IKBKBAS-AS, pcDNA3.1(+)-IKKβ 3′UTR, and pcDNA3.1(+)‑IKKβ 3′UTR-AS were linearized with the corresponding restriction enzymes that were used to clone the cDNAs at the 3′ end to prepare the template DNAs for in vitro transcription. The detailed steps are similar to a previous study [56].

### RNA-binding protein immunoprecipitation (RIP)

The RIP assays were performed using EZ-Magna RIP™ RNA-Binding Protein Immunoprecipitation Kits (Millipore) according to the manufacturer’s instructions. The gene‑specific primers for IKBKBAS were listed in Supplementary Table 3.

### Chromatin immunoprecipitation (ChIP)

A549 and HCC827 cells were treated with 40 ng/ml TNF-α (Peprotech, New Jersey, USA) for 12 h. The ChIP assay was carried out using EZ-Magna ChIP A/G (Millipore) with anti‑p65 antibody. Normal mouse IgG and anti-RNA Polymerase II was used as negative control and positive control, respectively. The target DNA fragments were detected by qPCR. The PCR products were also displayed by agarose gel electrophoresis. The primers used to detect target DNA fragments as well as antibody were listed in Supplementary Table 3.

### Statistical analysis

All data were statistically analyzed using GraphPad Prism 7 software (San Diego, CA, USA). Analysis of the differences between the two groups was determined by Student’s *t*-test. The correlation between IKBKBAS levels and IKKβ was assessed using Spearman’s correlation coefficient. A *p* value of <0.05 was considered statistically significant.

## Supplementary information

Supplementary figure and table legends

figureS1

figureS2

figureS3

figureS4

figureS5

Supplementary Table 1

Supplementary Table 2

Supplementary Table 3

## Data Availability

The datasets generated and/or analyzed during the current study are available from the corresponding author on reasonable request.
