# Supplementary material for: Novel cytoplasmic lncRNA IKBKBAS promotes lung adenocarcinoma metastasis by upregulating IKKβ and consequential activation of NF-κB signaling pathway
Source: Cell Death Dis. 2021 Oct 26;12(11):1004. doi: 10.1038/s41419-021-04304-4 (PMC8548314; doi:10.1038/s41419-021-04304-4)
Supplement: Supplementary file 1 — Supplementary figure and table legends [file 41419_2021_4304_MOESM1_ESM.docx]

**Supplementary figure legends**

**Fig. S1 Characterization of IKBKBAS in LUAD.**

(A）Volcano Plots showing the differentially expressed LncRNAs between 3 pairs of LUAD tissues in stage I and adjacent normal tissues.

(B) Venn diagram showing the intersection of differentially expressed lncRNA and mRNA in three pairs of specimens.

(C) Functional enrichment of the top 11 pathways of differentially expressed lncRNA data are shown as a bar chart.

(D) The 5′ and 3′ rapid amplification of cDNA ends (RACE) assays in H1299 cells to detect the whole sequence of lncRNA IKBKBAS. Left: gel electrophoresis images of PCR products from the 5′-RACE or 3′-RACE assays. Black arrows indicate product bands. Right: sequencing of PCR products indicated the boundary between the universal anchor primer and IKBKBAS sequences.

(E)The sequence of IKBKBAS was identical to LOC101929897 in GeneBanker by Blast.

(F) The scanning result of potential ORF and miRNA response elements (MREs) in IKBKBAS sequence predicted by RegRNA 2.0.

**Fig. S2 IKBKBAS promoted LUAD cells proliferation and metastasis both in vitro and in vivo.**

Overexpression of ectopic IKBKBAS and knockdown of IKBKBAS in H1299 cells were carried out for MTT assays (A), EdU assays (B), transwell assays (C), and plate colony formation assays (D).

(A and B) The effect of IKBKBAS on cell proliferation was evaluated by MTT assays and EdU assays.

(C) The effect of IKBKBAS on invasion and migration ability was evaluated by transwell assays.

(D) The effect of IKBKBAS on colonizing ability was determined by plate colony formation assays.

Data show mean ± SD, n ≥ 3, *P < 0.05, **P<0.01, ***P<0.001.

**Fig. S3 IKBKBAS is a positive regulator of NF-κB signaling pathway.**

(A) The relative expression of IKKβ in H1299 cells with ectopic expression of IKBKBAS was assayed by qRT-PCR.

(B) Western blot analysis for the expression of IKKβ, p-IKKβ，IκBα and p-IκBα in H1299 cells with ectopic expression of IKBKBAS.

Activity of NF-κB in A549 cells with ectopic expression of IKBKBAS and activity of NF-κB in HCC827 cells with knockdown of IKBKBAS were detected by EMSA.

(C) Activity of NF-κB in H1299 cells with ectopic expression of IKBKBAS or knockdown of IKBKBAS was detected by EMSA.

Data show mean ± S.E.M., n ≥ 3; *P< 0.05, **P<0.01, ***P<0.001.

**Fig. S4 Separation quality control for cell fractionation assays examined by Western blot.** H3 and GAPDH were used as separation quality standards.

**Fig. S5 MiR-512-5p is extremely down-regulated in LUAD cells and targets IKBKBAS.**

(A) Basal expression of miR-512-5p, miR-4741, miR-185-3p, miR-486-3p, miR-219b-5p, miR-4478 and miR-6805-5p in A549 and HCC827 cells were detected by qRT-PCR.

(B) The effect of miR-486-3p inhibitor or mimics on expression of IKBKBAS and IKKβ mRNA in A549 or HCC827 cells was assayed by qRT-PCR.

Data show mean ± S.E.M., n ≥ 3, *P < 0.05, **P<0.01，***P<0.001.

**Supplementary table legends**

**Supplementary Table 1 Differentially Expressed lncRNAs (Pass Volcano Plot).**

**Supplementary Table 2 Differentially Expressed mRNAs (Pass Volcano Plot).**

**Supplementary Table 3 Primers, probe and antibodies used in this study.**
