# Supplementary figures and images for: Novel cytoplasmic lncRNA IKBKBAS promotes lung adenocarcinoma metastasis by upregulating IKKβ and consequential activation of NF-κB signaling pathway
Source: Cell Death Dis. 2021 Oct 26;12(11):1004. doi: 10.1038/s41419-021-04304-4 (PMC8548314; doi:10.1038/s41419-021-04304-4)

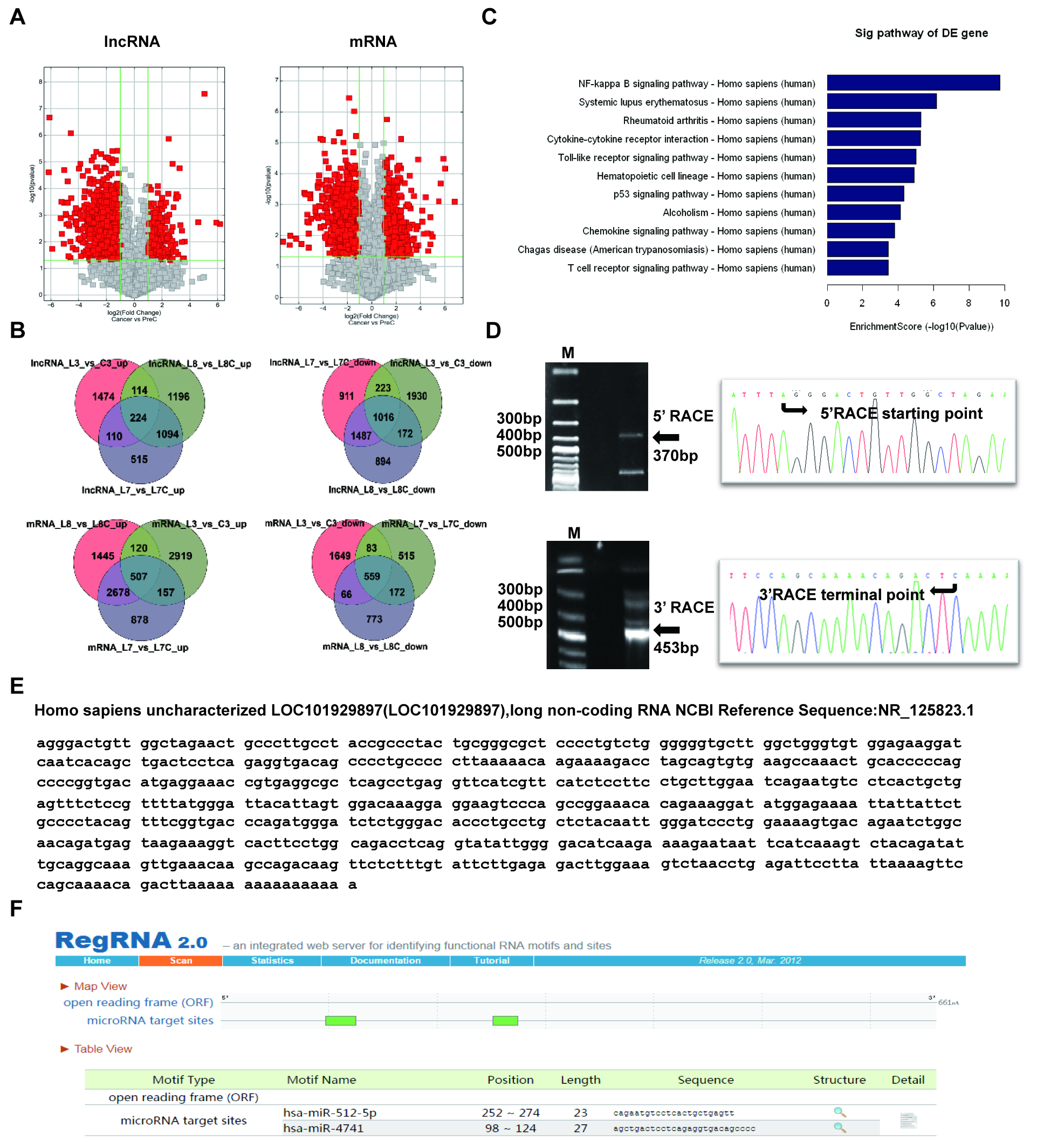

Supplement: Supplementary file 2 — figureS1 [file 41419_2021_4304_MOESM2_ESM.tif]

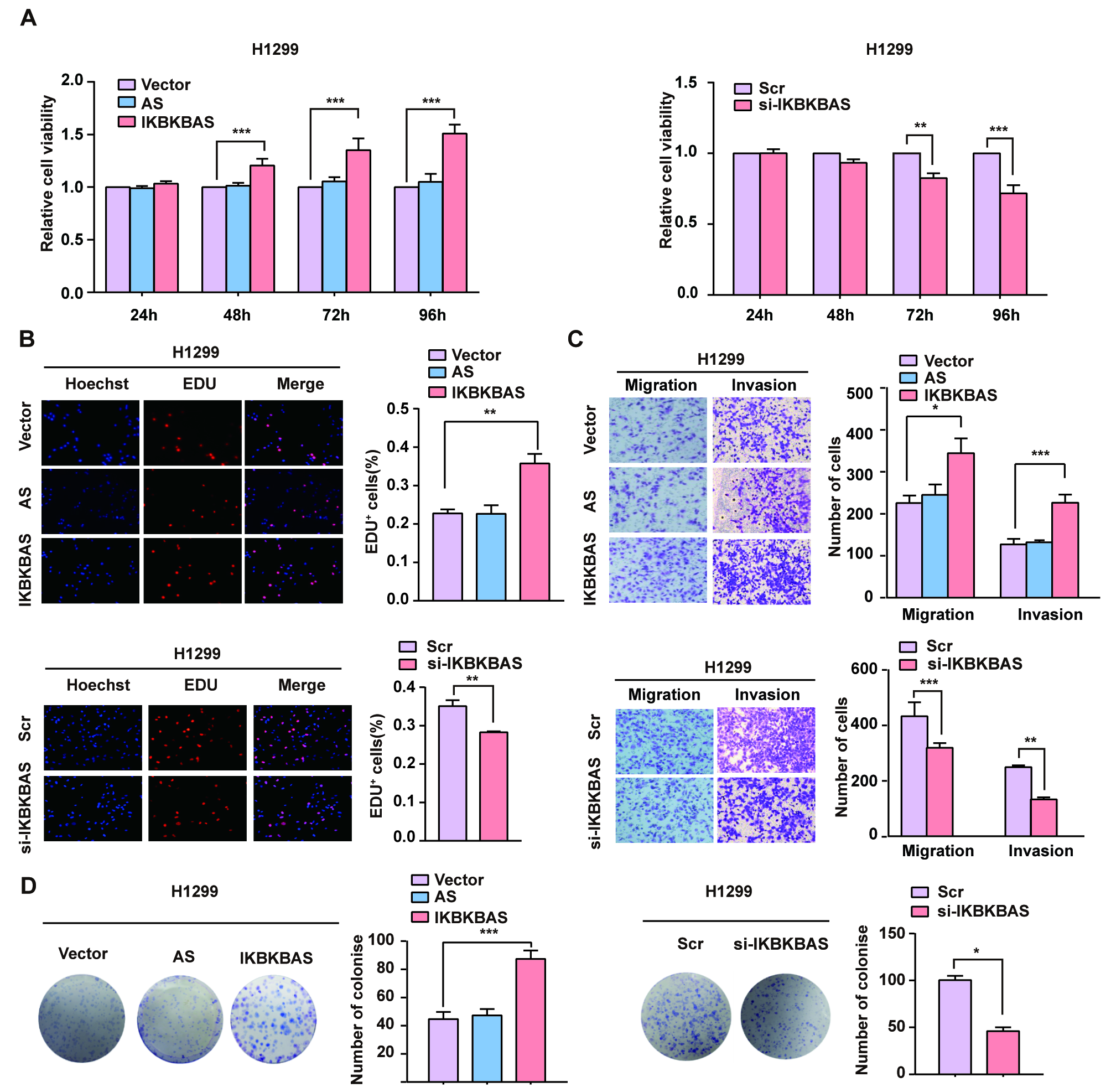

Supplement: Supplementary file 3 — figureS2 [file 41419_2021_4304_MOESM3_ESM.tif]

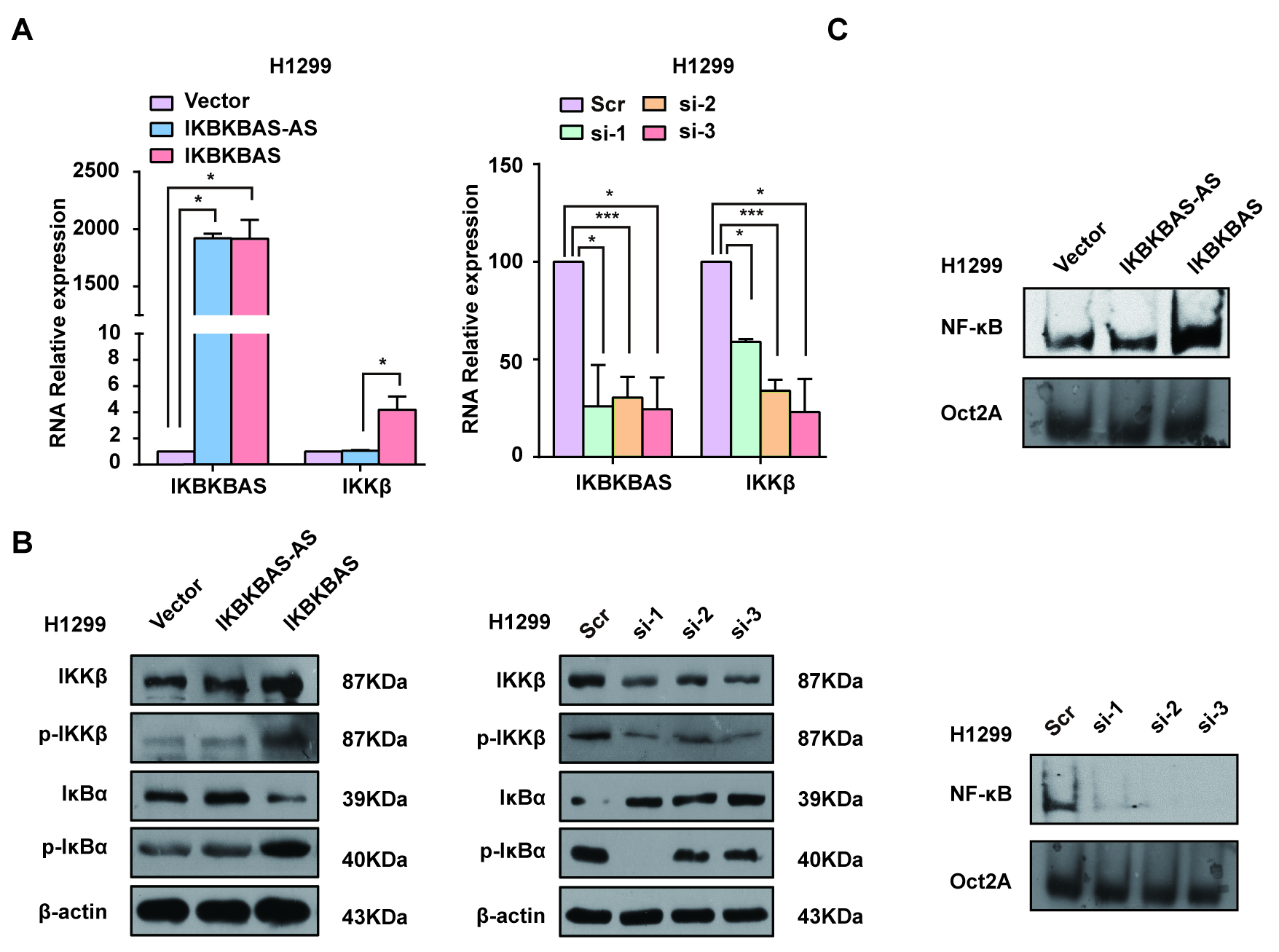

Supplement: Supplementary file 4 — figureS3 [file 41419_2021_4304_MOESM4_ESM.tif]

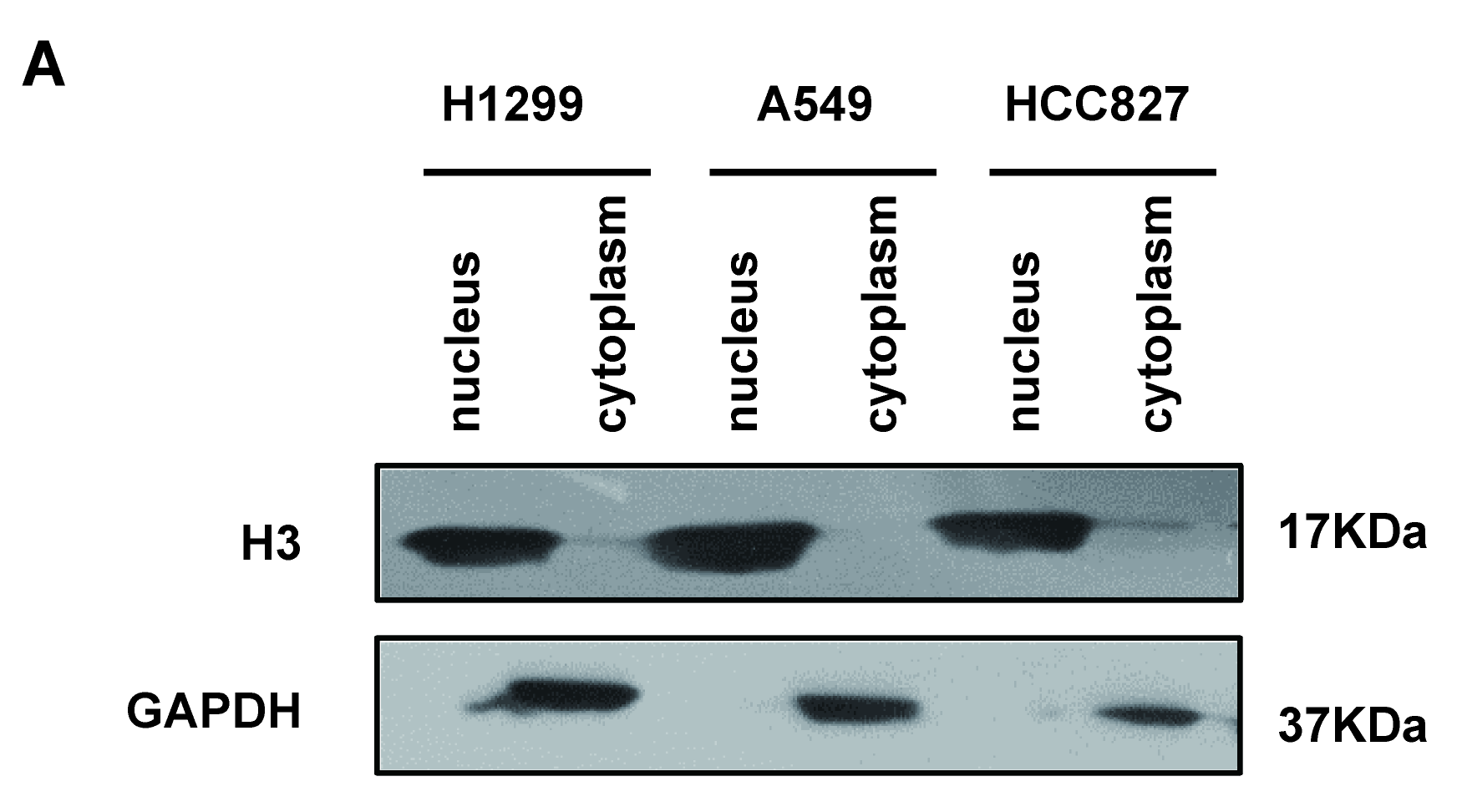

Supplement: Supplementary file 5 — figureS4 [file 41419_2021_4304_MOESM5_ESM.tif]

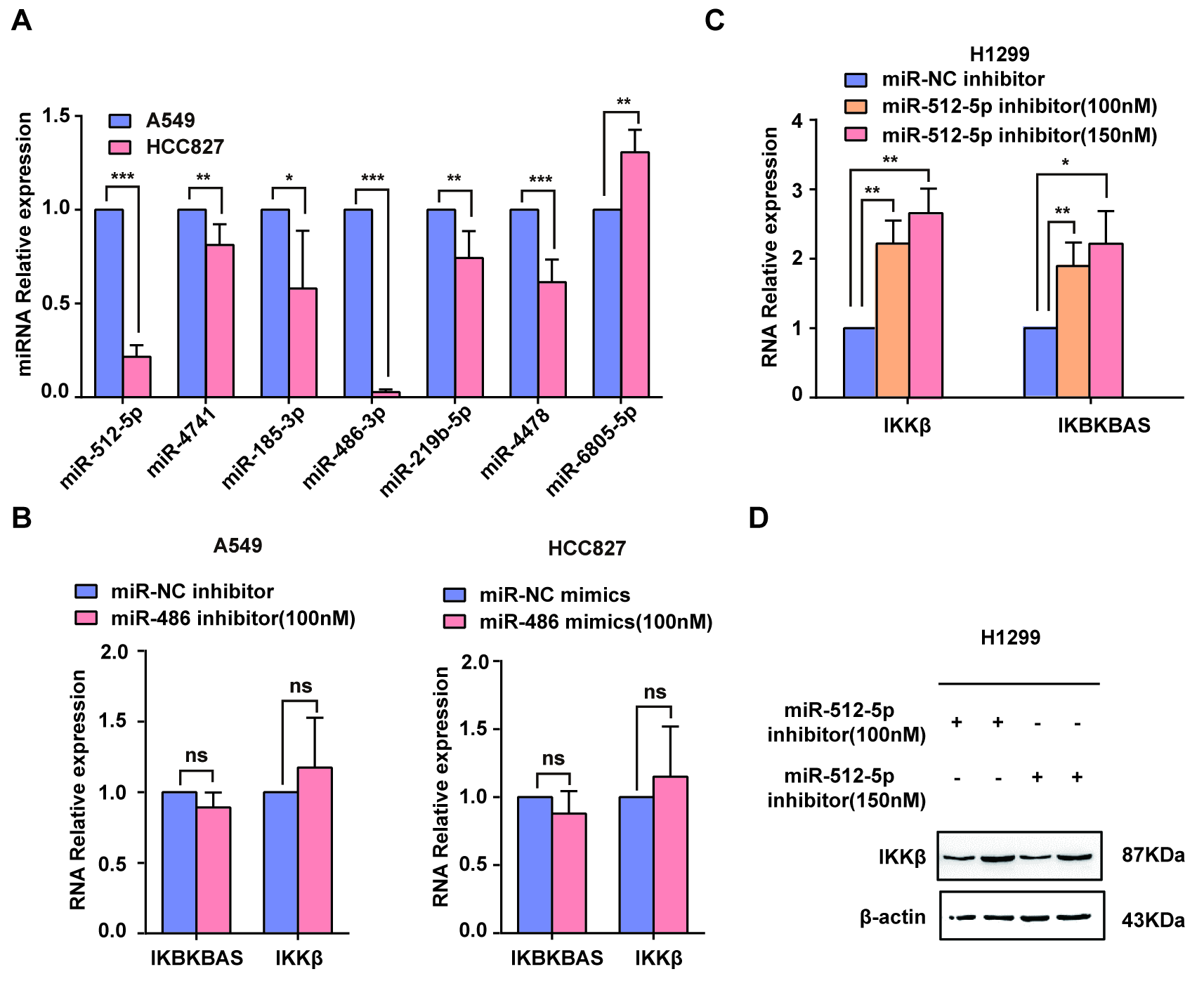

Supplement: Supplementary file 6 — figureS5 [file 41419_2021_4304_MOESM6_ESM.tif]
